# Lead Exposure and Associated Risk Factors among New Migrant Children Arriving in Greece

**DOI:** 10.3390/ijerph15061057

**Published:** 2018-05-23

**Authors:** Marsela Tanaka, Konstantinos Petsios, Stavroula K. Dikalioti, Stavroula Poulopoulou, Vassiliki Matziou, Stamatios Theocharis, Ioanna D. Pavlopoulou

**Affiliations:** 1 Pediatric Clinic, “P. & A. Kyriakou” Children’s Hospital, National and Kapodistrian University of Athens, Faculty of Nursing, Thivon & Levadeias str., Athens 11527, Greece; marstanaka@gmail.com (M.T.); vmatziou@nurs.uoa.gr (V.M.); idpavlop@yahoo.gr (I.D.P.); 2 Department of Nursing Research, Onassis Cardiosurgical Center, 356 Sygrou Avenue, Athens 17674, Greece; cpetsios@nurs.uoa.gr; 3 Department of Statistics, Athens University of Economy & Business, 76 Patission str., Athens 10434, Greece; spoulopo@gmail.com; 4 Department of Forensic Medicine and Toxicology, Faculty of Medicine, National and Kapodistrian University of Athens, 75 M. Asias str., Athens 11527, Greece; stamtheo@med.uoa.gr

**Keywords:** blood lead levels, children, immigrants, refugees

## Abstract

*Background:* This study aims to assess lead exposure and associated risk factors among newly arrived migrant (M) (immigrant and refugees) children in Greece and a matched control of native (N) children. *Methods:* A prospective, cross-sectional study was performed in an outpatient clinic of a tertiary children’s hospital. *Results:* From 2010 to 2014, 598 children (M/N: 349/249) with a mean age of 6.96 years old (range 1–14, SD 3.76) were enrolled. Blood lead levels (BLLs) ranged from 0.7 to 21 μg/dL in migrant and from 0.4 to 10 μg/dL in native Greek children. Elevated BLLs ≥ 5 μg/dL were detected in 27.7% of migrants and 1.2% of natives (*p* < 0.001). A significant association was found between EBLLs and childrens’ age (≤5 years) (OR: 1.8, *p*-value 0.02) and EBLLs with Asian origin (OR: 3.63, *p*-value 0.023). *Conclusion:* New migrant children presented with increased BLLs when compared to their age- and sex-matched controls. Younger age and Asian origin were significant risk factors associated with elevated BLLs among children. Early screening, secondary prevention, and regular follow-up could prove useful in this vulnerable population.

## 1. Introduction

Lead exposure and poisoning remains a preventable health issue for millions of children worldwide. There is sufficient evidence that lead exposure is associated with various long-term, adverse health outcomes, so several health organizations have issued prevention guidelines [1]. However, lead exposure continues to be a major concern, especially among the less privileged pediatric population [2,3].

The sources of lead contamination vary between developed [4,5] and developing countries [6,7]. Children are more likely to be affected, due to their physiology and behavior resulting in greater exposure, while they experience negative consequences due to increased absorption [8]. In response to the detrimental effects of lead poisoning, the Centers for Disease Control and Prevention (CDC) introduced a new reference blood lead level (BLL) of 5 μg/dL for children under the age of six years, highlighting, however, that no levels of lead may be considered “safe” for children [2].

Increased BLLs are more prevalent in immigrant and refugee children, which is interrelated to a number of contributing factors, such as exposure in their native country or during their journey of transition, as well as specific cultural habits or housing conditions that may persist in receptive countries [9]. Additional risk factors include younger age (<6 years old), low family income, living in old houses, passive smoking, maternal country of origin or exposure to lead during pregnancy [9,10,11]. Therefore, CDC recommends that all newly entered migrant children, between 6 months and 16 years of age, within 30–60 days of arrival to the United States should be screened for BLLs [9].

For over two decades, Greece has been facing an increased inflow of migrants, initially economic immigrants from Eastern European countries and recently refugees, mainly originating from the Middle East. According to the most recent Population and Housing Census data, the number of documented immigrants was calculated at 8.4% of the country’s total population [12]. The Ministry of Interior Affairs (2014) raised the number of migrants from third-country nations with valid stay permits up to 550.661, [13], whereas irregular migrants summed up to 428.935 amid 2010 and 2014 [14]. Among the aforementioned period, it has been estimated that one-third of the documented migrant population consists of children younger than 14 years old [15].

Considering the large influx of migrants in Greece and their possible lead exposure, it is important to identify the frequency and levels of lead exposure in the newly arriving pediatric population in order to create prevention strategies and policies.

The aim of the present study was to assess the prevalence of lead exposure among a population of migrant children, newly arrived in Greece, and compare this to a group of matched controls. A secondary objective was to identify possible risk factors associated with lead exposure in the group of migrants. 

## 2. Materials and Methods

### 2.1. Study Population 

In this prospective cross-sectional study, all migrant children between 1 and 14 years old, examined in a special outpatient clinic at “P. & A. Kyriakou” Children’s Hospital, within three months of their arrival in Greece, between May 2010 and March 2014 were enrolled in the study. The above population consisted of immigrant and refugee children who were either self-referred to obtain a green card (immigrants) or referred by non-governmental organizations for health status assessment before placement in shelters (refugees). Informed consent was obtained from all participants’ parents or guardians. The Institution Review Board of “P. & A. Kyriakou” Children’s Hospital approved the study protocol.

### 2.2. Data Collection

The guardians of all eligible children were informed of the study objectives and were interviewed in person using a structured questionnaire including sociodemographic data, past medical history, and date of entry in the country. Available medical, as well as travel documents, were also accessed. Interpreters were involved when necessary to achieve the best communication with children and parents. Children younger than 12 months or older than 14 years old, those unaccompanied, and those having entered the country for more than 3 months, were excluded. All children underwent a complete physical examination, including anthropometric measurements and growth assessment. Additionally, a group of healthy native children, attending as outpatients of the endocrinology, ophthalmology, and surgical departments of our hospital, matched by age and gender, were enrolled in the study as controls. Informed consent was obtained from all participants’ parents or guardians. The study was conducted in accordance with the Declaration of Helsinki, and the protocol was approved by the Institution Review Board of “P. & A. Kyriakou” Children’s Hospital (Reference number 145/19/05/10).

### 2.3. Laboratory Evaluation

Blood analysis with full blood count and ferritin serum levels was performed. The blood samples for measurement of BLLs were collected in ethylenediaminetetraasetic acid (EDTA) tubes, and stored at 4–6 °C in order to be used at a further stage. BLLs were measured by inductively coupled plasma–mass spectrometry (ICP-MS) following a 20-fold dilution with alkaline solution (concentrated nitric acid (2%), Triton X-100 (5%), and (NH_4_) 2HPO_4_ (2%)) using an Agilent 7500s-Agilent instrument with autosampler Cetas ASX 500 (Agilent Technologies, Waldbronn, Germany). The lower limit for detection of lead in blood was 0.03 μg/dL and that of quantitation was at 0.1 μg/dL.

Children were considered anemic if their hemoglobin levels were below the age-specific cutoffs according to WHO thresholds (Hb < 11 g/dL for ages 12–59 months; Hb < 11.5 g/dL for ages 5–11 years; Hb < 12 g/dL for 12–14 years) [16]. Low serum ferritin was defined at levels ≤12 ng/mL, and elevated BLLs (EBLLs) at levels ≥5 μg/dL [2].

Iron supplements were provided to all children with anemia or iron deficiency. Additionally, all immigrant and refugee children were provided with leaflets issued in their native language that included nutritional advice, information on lead poisoning effects, potential sources of contamination, and instructions on how to prevent exposure.

### 2.4. Glossary

The term “immigrants” (I), was used to describe children whose parents had a long-term residence permit, conforming to the terms of the International Organization for Migration (IOM) in the Glossary of Migration. The term “refugee” (R) was used to describe minors unable to return to their home country due to war or conflict, asylum seekers, and irregular entrants. Both immigrants and refugees were defined as “migrants” (M) [17].

### 2.5. Statistical Analysis

Children were distributed by immigrant or refugee status according to their demographic characteristics and medical history. The binary values of the BLLs (levels ≥5 μg/dL vs. <5 μg/dL) were used as the main endpoint of the analysis. The non-parametric Mann–Whitney and Kruskal–Wallis test was used for continuous variables, due to asymmetric data distributions, while for the categorical variables, the χ^2^ test (or Fisher’s test) was used. *p* < 0.05 was considered to indicate statistical significance. 

Multivariate logistic regression models were used to explore the risk factors of the EBLL occurrence. Τhe association between the child’s status (migrants versus native population) with the main health indicators: anemia (yes versus no) and ΕBLL (yes versus no) were investigated. The statistical analyses were conducted using the SPSS statistical package (IBM, v. 23.0, Chicago, IL, USA).

## 3. Results

### 3.1. Socio-Demographic Characteristics

A total of 351 migrant (190 refugees and 161 immigrants) and 249 native (*n*) children were enrolled from May 2010 to March 2014. Two refugee children were subsequently excluded due to an incomplete evaluation of their health status. Table 1 illustrates the main demographic and screening characteristics of the study population. As shown, 18.9% of migrant and 13.3% of native children (*p* = 0.041) presented with anemia, and 17.5% and 6.4%, respectively, were iron-deficient (*p* ≤ 0.0001). 

### 3.2. Blood Lead Levels

BLLs in both groups ranged from 0.7 to 21.03 μg/dL (0.7–21 μg/dL in migrants and 0.4–10 μg/dL in natives), and mean values of BLLs of all children (both natives and migrants) were 2.9 ± 2.4 μg/dL, with a median of 2.2 (0.4–21) μg/dL. Among migrant children, those belonging to the immigrant subgroup had mean BLLs of 4.3 μg/dL and a median of 3.7 μg/dL; the refugee mean was 3.8 μg/dL, and the median was 3.4 μg/dL. In contrary to those, mean BLLs found in the native participants was 1.3 μg/dL (median: 1.1 μg/dL). Moreover, the mean age of all children with EBLLs was 4.89 ± 2.26 years (range 12 months–14 years).

In Table 2, the distribution of low (less than 5 μg/dL) and elevated BLLs (equal to or greater than 5 μg/dL) among different groups are presented. In three out of 598 children (0.005%) the evaluation of BLLs was not possible. It is noteworthy that 27.7% of migrant children presented EBLLs, in contrast to 1.2% of natives (*p*-value ≤ 0.0001). Among migrant children with EBLLs, the majority (93.8%) presented lead levels between 5 and 10 μg/dL. A percentage of 6.2% presented with EBLLs >10 μg/dL (max 21.3 μg/dL). In contrast, only three children from the group of natives showed EBLLs (two with BLLS from 5 to 10 μg/dL, and one with >10 μg/dL). Interestingly, lead exposure prevalence was slightly higher in immigrant children (32.3%) as compared to refugees (25 %), *p* = 0.05.

The vast majority of migrant children with EBLLs originated from Asia (*n* = 90, 93.8%), mainly from Afghanistan (44.4%) and Bangladesh (23.3%), while the rest were newcomers from Pakistan (17.8%), India (8.9%), Syria (2.2%), China (1.1%), Philippines (1.1%), or Iran (1.1%). Children from Africa originated from Egypt (2.1%), Morocco (1.05%), and DR Congo (1.05%), while those from Europe were migrants from Albania and Georgia (1.05%).

### 3.3. Risk Factors 

A logistic regression analysis of the possible risk factors associated with EBLLs is presented in Table 3. The factors related to EBLLs were a younger age (1–5 years old) (OR = 1.8 (1.10–2.93), *p*-value 0.02) and the geographic area of origin (Asia, OR = 3.63 (1.20–10.97), *p*-value 0.023). The association of migration status with EBLLs was of marginal statistical significance (Immigrants, OR = 5.94 (0.95, 37.23), *p*-value 0.057). Univariate analysis showed that immigration status as independent risk factor was not associated with EBLLs (*p*-value = 0.092). Although a relation between anemia, iron depletion, and EBLLs was identified following multiple logistic regression, these variables were not statistically significant (OR = 1.28, *p*-value 0.436; OR = 1.39, *p*-value 0.30). 

## 4. Discussion

The present study is the first to provide evidence of BLLs in newly arriving migrant children in Athens, Greece. Overall, 27.7% of new migrant children presented laboratory evidence of lead exposure, significantly higher than native children (1.2%), while in 6.2% of them BLLs exceeded levels of 10 μg/dL. A younger age and being of Asian origin were identified as significant risk factors for elevated BLLs in our study group. Assessment of lead exposure upon arrival of migrant children and subsequently after their resettlement in the host country to identify ongoing exposure should be considered as a public health priority since children at risk show no clinical signs and are only identified during screening.

BLLs in new migrant participants were significantly higher than those measured in the native population. This is in accordance with data from the United States [18,19,20], Canada [10], and Europe [21], where newly arrived overseas children are documented with increased BLLs compared to native children. Newly arrived (<72 months old) refugee children in Minnesota of the USA (2004–2005) were found to have a 14 times greater prevalence of lead exposure than their native peers [9]. Eisenberg et al (2011) stated that the prevalence of EBLLs (10 μg/dL or higher) in newly arrived refugee children was 16% in comparison to 1.4% of the US population living in Massachusetts [18].

Anemia and iron deficiency were common findings among children with lead exposure. Lead impairs heme synthesis and increases red blood cell destruction, causing anemia [22]. EBLLs, especially levels exceeding 40 μg/dL, have been associated with anemia [23]. However, anemia has also been described among individuals with lower BLLs, even at 10–19.9 μg/dL [22]. Indeed, we observed an association between children with anemia and those with EBLLs (66/99; 18.9%, *p*-value 0.041) in the Fisher’s exact test, but this was not statistically significant following multivariate analysis. These results are in accordance with previous studies that found no correlation between BLLs and anemia [21,24,25]. Moreover, multivariate analysis showed no association between iron deficiency and EBLLs. In literature, there are studies in line with these results [25,26], while others support that, even low BLLs are associated with iron deficiency [27,28]. Iron supplements are found to decrease BLLs and CDC recommends correcting iron deficiency in this group of vulnerable children [2,9,29].

Multivariate analysis revealed younger age and geographic area of origin as significant risk factors for EBLLs, whereas migration status was only marginally associated. Children 1–5 years old were more likely to have increased levels of lead. We assume that younger children were more susceptible to lead exposure due to hand-to-mouth behavior that may lead to increased exposure in a lead-abundant environment [30]. There is evidence of adverse effects, especially in preschool children, related to the poor hygiene practices and uncontrolled levels of lead in their residential environment, affecting the development of their nervous system and behavior [1,30,31]. In a previous study in Massachusetts and New Hampshire, the median age of those with elevated BLL on repeat testing was 4.9 years (range 14 months–13 years) [32]. In our case, the mean age of children with EBLLs was 4.89 ± 2.26 years (range 12 months–14 years), which is in accordance with previous results, indicating this age group (1–5 years old) as the most vulnerable to lead exposure [19].

The highest BLLs were detected in children originating from Asia, particularly from South Central Asia, namely Afghanistan and Bangladesh. In a sample of 1406 refugee children screened for BLLs in Washington, USA, (2013–2014), those from Afghanistan (50%) presented higher percentage of EBLLs (≥5 µg/dL), compared to those from Somalia (15%), Burma (16%), and Iraq (11%). This was related to the exclusive use of leaded gasoline [31]. Similarly, Bangladesh has been described as a country of high lead pollution [6]. According to a survey conducted in New York, in a sample of 230 South Asian adults and children, Bangladeshi and Indian population showed higher BLLs, compared to others [33]. This finding was associated with a number of reasons, such as traveling in developing countries and the use of local products. Mitra et al. reported that, in Bangladesh, among 345 children (6 months–12 years old), 39% were detected with BLLs ≥ 10 µg/dL [6]. Finally, Pakistan is also characterized as a country of elevated risk for lead exposure [34,35].

Both immigrant and refugee children are described as a population at risk for EBLLs. Possible explanations include environmental exposure, living conditions, eating habits, parental occupation, socio-economic status, cosmetic use, and (mouth and hand) hygiene behavior in the country of origin [2,9]. It must be emphasized that migrant children may experience suboptimal living conditions in the receptive country, leading to continued lead exposure. These include living in old overcrowded houses and burning fossil fuels and waste for heat, along with use of lead-containing traditional remedies, foods, ceramics, and utensils [2,36]. It is noteworthy that the proportion of children with EBLLs was higher among immigrants, but this was not statistically significant. However, our data collection occurred between 2010 and 2014, when most immigrants originated from Bangladesh, Pakistan and India, whereas the population of refugees originated from Afghanistan. All the aforementioned countries have been described as increased risk for lead exposure [6,34,37]. It is evident, that no firm conclusions can be drawn by this finding, as results might differ according to the proportion of ethnicities or additional risk factors among the two subpopulations of migrants. 

This study has some limitations. The reported results are restricted to a subpopulation of immigrants and refugees arriving in Athens and, therefore, must be interpreted accordingly. Additionally, the fact that unaccompanied refugee children, not able to provide informed consent, were excluded from our study might affect the overall estimates, as this group may be more susceptible to lead exposure. It must be noted though that at that time their number was small (*n* = 15).

Furthermore, due to language barriers, we lack detailed records with regard to additional factors possibly related to EBLLs in migrants, so we could not investigate respective associations on the reported difference of BLLs between the group of migrant and native children. Our study population was chosen to evaluate solely newly arrived migrant children to collect baseline information. Thus, we were unable to assess the possibility of continued lead exposure due to personal habits or living conditions after migration, which could be an essential factor for ongoing exposure and harm to those children residing in Greece for extended periods of time. 

Finally, the gender-distribution among the two groups of native versus migrant children is different with more girls among the subgroup of Greek children. Girls are generally known to experience lower lead exposure, so this discrepancy presumably exaggerates the BLL difference between the two groups. 

Despite the above limitations, the study has some strong points. It is the first study to document the laboratory evidence of lead exposure and certain associated risk factors in a subpopulation of new immigrant and refugee children arriving in Greece. Since the prevention of lead exposure in children is essential considering its possibly irreversible effects, if present, and the fact that there are no “safe” blood levels, our findings can guide the development of effective prevention strategies addressing this vulnerable population.

## 5. Conclusions

We found that a considerable proportion of newly arriving migrant children in Greece present laboratory evidence of lead exposure that is significantly higher than their native counterparts. A younger age and being of Asian origin were significant risk factors for EBLLs. Screening of all migrant children soon after their arrival, especially the youngest ones and those originated from South Central Asia, appears essential. Additional data regarding lead exposure of migrant children upon arrival and after resettlement from host countries in Europe are necessary to provide evidence-based screening recommendations. Early screening, secondary prevention, and regular follow-up could prove useful in this vulnerable population.

## Figures and Tables

**Table 1:** Characteristics and laboratory evaluation of new migrant (*n* = 349) and native (*n* = 249) children.

	Immigrant (*n* = 161)	Refugees (*n* = 188)	Migrant ^a^ (*n* = 349)	Native Controls (*n* = 249)	Total (*n* = 598)	*p*-value ^#^
	(*n*, %)					
Gender						
Male/Female (%/%)	104/57 (64.6/35.4)	107/81 (56.9/43.1)	211/138 (60.5/39.5)	129/120 (51.8/48.2)	340/258 (56.9/43.1)	0.022 ^1^
Age (years)						
mean±SD	6.25 ± 3.69	7.81 ± 3.69	7.09 ± 3.77	6.78 ± 3.74	6.96 ± 3.76	0.324 ^2^
Median (range)	5 (1–14)	8 (1–14)	7 (1–14)	5.5 (1–14)	6 (1–14)	
Age group (years)						
1–5	89 (55.3)	58 (30.9)	147 (42.1)	129 (51.8)	276 (46.2)	0.012 ^1^
6–14	72 (45.3)	130 (69.1)	202 (57.9)	120 (48.2)	322 (53.8)	
Geographic area of origin						
Europe	30 (18)	0 (0)	30 (8.6)	249 (100)	279 (46.7)	≤0.0001 ^3^
Asia	113 (70.2)	173 (92)	286 (81.9)	0 (0)	286 (47.8)	
Africa	18 (11.2)	15 (8)	33 (9.5)	0 (0)	33 (5.5)	
Social Security						
NOHSP ^b^	73 (45.3)	0 (0)	73 (20.9)	249 (100)	322 (53.8)	≤0.0001 ^3^
Refugee card	0 (0)	188 (100)	188 (53.9)	0 (0)	188 (31.4)	
M. E. Card ^c^	15 (9.3)	0 (0)	15 (4.3)	0 (0)	15 (2.5)	
Uninsured	73 (45.3)	0 (0)	73 (20.9)	0 (0)	73 (12.2)	
BMI ^d^, (in percentiles)						
<5th	29 (18)	26 (13.8)	55 (15.8)	25 (10)	80 (13.4)	0.001 ^3^
5th–15th	26 (16.1)	38 (20.2)	64 (18.3)	25 (10)	89 (14.9)	
15th–85th	82 (50.9)	104 (55.3)	186 (53.3)	147 (59)	333 (55.7)	
85th–95th	12 (7.5)	9 (4.8)	21 (6)	31 (12.4)	52 (8.7)	
>95th	12 (7.5)	11 (5.9)	23 (6.6)	21 (8.4)	44 (7.4)	
Anemia ^e^						
No	144 (89.4)	139 (73.9)	283 (81.1)	216 (86.7)	499 (83.4)	0.041 ^1^
Yes	17 (10.6)	49 (26.1)	66 (18.9)	33 (13.3)	99 (16.6)	
Pb **^,f^ (≥5 μg/dL)						
No	109 (67.7)	141 (75.0)	250 (72.3)	246 (98.8)	496 (83.4)	≤0.0001 ^1^
Yes	52 (32.3)	47 (25.0)	99 (27.7)	3 (1.2)	99 (16.6)	
Iron deficiency *^,^^g^ (≤12ng/dL)						
No	127 (78.9)	161 (85.6)	288 (82.5)	233 (93.6)	521 (87.1)	≤0.0001 ^1^
Yes	34 (21.1)	27 (14.4)	61 (17.5)	16 (6.4)	77 (12.9)	

^1^ Fisher’s test; ^2^ Mann–Whitney Test; ^3^ Pearson’s Chi Square Test. ^a^ Migrants: Refugees and Immigrants; ^b^ NOHSP: National Organisation for Healthcare Services Provision; ^c^ M.E. card: Medical Eligibility card; ^d^ BMI: Body Mass Index; ^e^ Definition of anemia by age group: Hb < 11 mg/dL (12–59 months); Hb < 11.5 mg/dL (5–11 years); Hb < 12 mg/dL (12–14 years); ^f^ Pb: Blood Lead Levels; * *n* = a total of 598 children were tested; ** *n* = a total of 595 children were tested; ^#^ *p*-values refer to natives versus migrants comparisons.

**Table 2:** Blood lead levels (BLLs) in migrants and native children.

	Pb ^a,^*		Total	*p*-Value
	<5 (μg/dL) *n*, (%)	≥5 (μg/dL) *n*, (%)		
Age group, (years)				
1–5	222 (44.8)	54 (54.5)	276 (46.4)	0.047 ^1^
6–14	274 (55.2)	45 (45.5)	319 (53.6)	
Gender				
Male/Female	279/217 (56.3/43.8)	61/38 (61.6/38.4)	340/255 (57.1/42.9)	0.191 ^1^
Migration status				
Migrant	250 (50.4)	96 (97)	346 (58.2)	≤0.0001 ^1^
Native	246 (49.6)	3 (3)	249 (41.8)	
Geographic area of origin				
Europe	274 (55.2)	5 (5.1)	279 (46.9)	≤0.0001 ^1^
Asia	196 (39.5)	90 (90.9)	286 (48.1)	
Africa	26 (5.2)	4 (4)	30 (5)	
Anemia **				
No	423 (85.2)	73 (73.7)	496 (83.4)	0.005 ^1^
Yes	73 (14.7)	26 (26.3)	99 (16.6)	
Iron deficiency				
No	443 (89.3)	76 (76.8)	519 (87.2)	0.001 ^1^
Yes	53 (10.7)	23 (23.2)	76 (12.8)	
BMI ^b^, (in percentiles)				
<5th	59 (11.9)	21 (21.2)	80 (13.4)	0.003 ^2^
5th–15th	68 (13.7)	20 (20.2)	88 (14.8)	
15th–85th	288 (58.1)	43 (43.4)	331 (55.6)	
85th–95th	48 (9.7)	4 (4)	52 (8.7)	
>95th	33 (6.7)	11 (11.1)	44 (7.4)	

^1^ Fisher’s test; ^2^ Pearson’s Chi Square Test; ^a^ Pb: Blood lead levels; ^b^ BMI: Body Mass Index; * *n* = a total of 595 children were tested; ** Definition of anemia by age group: Hb <11 mg/dL (12–59 months); Hb <11.5 mg/dL (5–11 years); Hb <12 mg/dL (12–14 years).

**Table 3:** Multiple logistic regression derived odds ratios (and 95% confidence intervals) for the risk factors for elevated blood lead levels (EBLLs).

	Elevated Blood Lead Levels *		*p*-Value
	(≥5 μg/dL vs. <5 μg/dL)		
	OR	95% CI.	
Migration status (Reference category: Natives)			
Immigrants	5.94	(0.95,37.23)	0.057
Refugees	3.15	(0.47,21.16)	
Age Groups (years) (Reference category: 6–14)			
(1–5 years)	1.8	(1.10, 2.93)	0.02
Geographic area of origin (Reference category: Africa)			
Europe	0.37	(0.06, 2.21)	0.273
Asia	3.63	(1.20, 10.97)	0.023

* *n* = 595 children tested.
